# *Porphyromonas gingivalis* Fimbria-Induced Expression of Inflammatory Cytokines and Cyclooxygenase-2 in Mouse Macrophages and Its Inhibition by the Bioactive Compounds Fibronectin and Melatonin

**DOI:** 10.5402/2012/350859

**Published:** 2012-04-01

**Authors:** Yukio Murakami, Mamoru Machino, Seiichiro Fujisawa

**Affiliations:** Division of Oral Diagnosis, Department of Diagnostic and Therapeutic Sciences, Meikai University School of Dentistry, 1-1 Keyakidai, Sakado-City, Saitama 350-0283, Japan

## Abstract

*Porphyromonas gingivalis* (Pg) fimbriae, in addition to lipopolysaccharide, are involved in the pathogenesis of periodontal disease. At the same time, bioactive compounds such as fibronectin (FN) and melatonin in saliva and gingival crevicular fluid have been reported to exert a preventive effect against periodontitis. Here, we review current knowledge regarding the potent inhibitory effects of FN and melatonin against Pg fimbria-induced induction of proinflammatory cytokines, cyclooxygenase-2 (COX-2) expression, and NF-kappa B activation in mouse macrophages and discuss their possible clinical application for prevention of periodontal diseases induced by oral bacteria.

## 1. Introduction

The systemic dynamic proinflammatory cellular response to localized periodontal bacteria can sometimes lead to widespread organ damage or even death. *Porphyromonas gingivalis* (Pg) is a key organism associated with periodontal destruction in patients with adult periodontitis. Many researchers (such as Kadowaki et al. [1], Bainbridge and Darveau [2], Laine et al. [3], and Davey et al. [4]) have shown that Pg produces several virulence factors, including outer membrane vesicles, adhesins, lipopolysaccharide (LPS), hemolysin, and proteinases. Renshaw et al. [5] have also shown that changes in the innate immune response with aging include impairment of the capacity to recognize and respond to periodontal pathogens and that these changes may partly result from reduced expression of cell surface receptors, such as Toll-like receptors (TLRs). TLRs are associated with the recognition of Pg components such as LPS and fimbriae, which in turn leads to aspects of periodontal disease such as cytokine and chemokine production and oral bone loss [4, 6, 7]. It is well known that the adhesion of Pg to host cells is a prerequisite step in the pathogenesis of Pg-induced periodontal disease. Pg binds to and invades epithelial cells, and the fimbriae are known to be intrinsically involved in the first step of this process. Fimbriae are peritrichous filamentous appendages, whereas LPS is a major component of the outer membrane of Pg and is capable of host activation, mediating the adhesion of bacteria to both host cells and a variety of oral substrates and molecules [8]. Pg LPS in particular is thought to stimulate the production of both catabolic cytokines and inflammatory mediators including arachidonic acid metabolites such as prostaglandin E_2_. Such cytokines and inflammatory mediators promote the release of tissue-derived enzymes, the matrix metalloproteinases (MMPs), which are destructive to extracellular matrix and bone [4, 6, 7]. Pg fimbriae are also implicated in the pathogenesis of periodontal disease [9, 10]. Davey et al. have reported that bacterial fimbriae activate a proinflammatory response in the endothelium through distinct TLRs [4]. Hanazawa et al. have investigated the possible activation of mouse peritoneal macrophages by 43-kDa Pg fimbriae, including gene expression and production of interleukin-1, as well as bone resorption [9, 11]. Umemoto and Hamada have also indicated that two microbial components of Pg fimbriae, the major (41-kDa) and minor (67-kDa) fimbriae, may be involved in inflammatory responses [12]. The major fimbriae induce the expression of inflammatory cytokines, including TNF-alpha and IL-1beta [7], whereas the minor fimbriae induce TNF-alpha, IL-1beta, and IL-6 production in human monocyte cell lines and murine peritoneal macrophages [7, 13]. Hajishengallis et al. have shown that the receptors involved in the inside-out proadhesive pathway (CD14, TLR2, and CD11b/CD18) are important for mediating Pg internalization within macrophages and also that Pg appears to proactively modulate beta2 integrin adhesive activity for intracellular uptake [14]. In addition, they have reported interaction of Pg with complement receptor-3 (CR3; CD11b/CD18) in monocytes/macrophages [15]. Previously, we have suggested the importance of beta2 integrin (CD11/CD18) as a cellular receptor of Pg fimbriae at the initiation stage of periodontal disease pathogenesis [16]. Active Pg invasion of human vascular cells and stimulation of adhesion molecules involved in the recruitment of leukocytes to sites of inflammation by Pg may also play a role in the pathogenesis of systemic inflammatory diseases associated with this microorganism, including atherosclerosis [17].

Prostaglandin E_2_, a vasoactive eicosanoid produced by monocytes and fibroblasts, induces bone resorption and secretion of MMP. A causal link between periodontal inflammation and elevated levels of PGE_2_ in gingival tissue and crevicular fluid has been demonstrated by many researchers [18–20]. Howell and Williams [18] have reported the importance of eicosanoids in the pathogenesis of periodontal disease and reviewed the beneficial effects of nonsteroidal anti-inflammatory drugs (NSAIDs) in both animal models and humans with periodontitis [19, 20]. NSAIDs are known to act by downregulating eicosanoid synthesis.

We have extensively investigated the Pg fimbria-induced expression of inflammatory cytokines and COX-2 in mouse macrophages and the inhibitory effects of the bioactive compounds, FN and melatonin, on this expression [21, 22]. Here, we present the results of our previous experiments and the extent of current knowledge regarding the possible preventive effects of bioactive compounds such as FN and melatonin against periodontal disease.

## 2. Fibronectin

FN is a multifactor glycoprotein found in insoluble form in blood, saliva, and other body fluids [23, 24], where it interacts with several extracellular matrix (ECM) components such as collagen and laminin. These molecules usually provide structural support to mammalian cells and act as a scaffold for cell adhesion and retention of growth factors. Several previous studies have shown that these proteins are able to bind to various bacterial species, including *Streptococcus *spp., *Escherichia coli*, and *Treponema *spp. [25–28] and that fimbriae play a major role in this process [29, 30]. In general, bacterial fimbriae are considered to be a major virulence factor involved in bacterial adhesion to and invasion of host cells. Current knowledge suggests that the matrix proteins act as an inhibitory barrier to bacterial adhesion. However, Pg also binds to the extracellular matrix [31–33]. We have previously demonstrated that the fimbriae of Pg bind to FN, mediating Pg infection of host cells [34]. Moreover, Kawata et al. have observed that fimbria-stimulated bone resorption is inhibited by pretreatment with FN [35]. In addition, we have observed that FN is negatively regulated by fimbria-stimulated endogenous interleukin-6 and that the amount of FN in gingival crevicular fluid is decreased in patients with severe periodontal disease [36]. These observations suggest that FN might play a functional role as a negative regulator of fimbria-mediated pathogenesis in the initiation and development of chronic periodontal disease. On the other hand, the attachment of alphavbeta3- and alpha5beta1-integrin-overexpressing CHO cells to the polystyrene culture dishes in the presence of their ligand ECM (vitronectin and FN) proteins, and the binding of vitronectin and FN to these cells were inhibited by the fimbriae and *P. gingivalis* cells, respectively. These results suggest that fimbriae compete with ECM proteins for these integrins and suppress integrin/ECM protein-related cellular functions [37].

**Figure 1:**
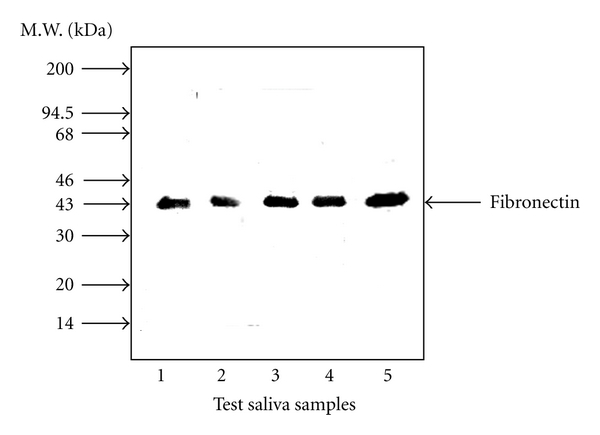
Binding of fibronectin in saliva to *P. gingivalis* fimbrillin. The purified fimbriae (10 *μ*g/mL) were subjected to sodium dodecyl sulfate polyacrylamide gel electrophoresis (SDS-PAGE) in 12.5% polyacrylamide gel, and then the separated samples were transferred to a PVDF membrane. The membrane was then treated for 2 h at 37°C with saliva from healthy subjects. Salivary FN that became bound to the fimbriae was analyzed by overlay Western Blotting with anti-FN antibody and visualized using horseradish-peroxidase (HRP-) conjugated goat anti-rabbit IgG. Three independent experiments were performed, and similar results were obtained (Copyright Wiley-Blackwell).

The results of our previous study of the binding of FN in saliva to Pg fimbriae using Western Blot assay are shown in Figure 1. FN in saliva of a healthy subject became bound to 43-kDa Pg fimbrillin [21]. In that study, the FN content of saliva was examined in healthy subjects (*n* = 48) and patients with adult periodontal disease (*n* = 55). The FN level in the saliva of the patients was significantly lower than that in the healthy subjects (*P* < 0.01). Talonpoika et al. [38] showed that both intact FN and FN fragments were present in gingival crevicular fluid of healthy subjects and patients with periodontal disease. A large proportion of FN was in degraded form at diseased sites exhibiting clinical signs of inflammation and with pockets at least 4 mm deep, than in healthy or treated sites, and FN was degraded into smaller peptide fragments at diseased than at treated sites. This suggested that FN is partially degraded in both healthy and diseased periodontium and that the degree of FN degradation increases with periodontal inflammation and decreases with periodontal treatment.

The existence of FN fragments was previously reported to be involved in chronic inflammatory diseases including periodontitis and arthritis [39]. Huynh et al. reported that FN fragments in gingival cervicular fluids derived from possible infection, inflammation, and wounding were 40, 68, and 120 kDa in size. These fragments may be derived from the decomposition of FN by proteases such as MMP in gingival crevicular fluid [40], and it was suggested that FN fragments may be used as a marker of periodontal disease status. Kapila et al. have shown that FN and specific FN fragments can differentially induce the expression of proteinases in periodontal ligament cells, subsequently contributing to tissue degradation during periodontal disease, wound healing, and maintenance of the extracellular matrix in periodontal tissues [41]. In addition, activities of the alternatively spliced V region and high-affinity heparin-binding domain of FN are mediated by transcriptionally dependent decreases in p53 and c-Myc, thus inducing apoptosis in human primary cells through the novel alternative pathway [42]. A proapoptotic FN matrix induces ubiquitination and degradation of p53 in the proteasome [43]. Thus, FN molecules contribute to the novel mechanism of apoptosis associated with inflammatory disease.

Recently, two types of Pg fimbrial structure have been described: the so-called major fimbria encoded by the *fimA* gene and minor fimbria encoded by the *mfa1* gene [12, 44]. Pg fimbriae recognize various host cell substances [16, 45, 46]. The fimbrillin subunit (FimA) constitutes the main structural component, but accessory proteins such as FimC, FimD, and FimE located downstream of FimA are necessary for virulence against host cells [46]. Pierce et al. have demonstrated that FimCDE components, such as CXC-chemokine receptor 4, FN, and type 1 collagen, cooperate and confer critical adhesive and virulence properties on Pg fimbriae [47]. Thus, binding to receptors on host cells or extracellular matrix components such as FN may be required in order for both major and minor fimbriae to express periodontal pathogenicity. It will be necessary to investigate novel structural proteins of fimbriae, such as FimCDE, as potential targets for inhibitory molecular intervention against Pg infection so that FN can function more effectively as a barrier to periodontal destruction.

## 3. Melatonin

 Melatonin is the major secretory product of the pineal gland and is mostly associated with regulation of the circadian dark/light rhythm of the human body [48, 49]. This molecule is also produced in other tissues such as the retina, bone marrow, gastrointestinal tract, gonads, and immune system [50–52]. Melatonin has been recently recognized as a potent antioxidant and immunomodulator and is considered to be an important natural oncostatic agent [48, 49]. Melatonin passes into saliva by passive diffusion from the bloodstream. Cutando et al. [53, 54] and Gómez-Moreno et al. [55, 56] have focused on melatonin and its preventive effects against oral cavity disorders and also in oxidative stress-related oral diseases and periodontal inflammation.

COX-2 is the key enzyme that catalyzes the two sequential steps in the biosynthesis of prostaglandins (PG)s from arachidonic acid. COX-2, the inducible isoform of COX, plays a critical role in the inflammatory response, and its overexpression has been associated with several pathologies including neurodegenerative diseases and various types of cancer. Mayo et al. have investigated the suppressive effect of melatonin and its metabolites on the activities of COX-2 and inducible nitric oxide synthase (iNOS), using LPS-activated RAW264.7 macrophages as a model [57]. In addition, Deng et al. have shown that melatonin, but not tryptophan or serotonin, time- and concentration-dependently inhibits the LPS*-*induced protein levels and promoter activities of COX*-*2 and iNOS in RAW264.7 cells [58]. Note that melatonin, like serotonin and tryptophan, is an indole derivative. Furthermore, Noguchi et al. have suggested that COX-2-dependent exogenous PGE_2_ downregulates IL-1alpha-induced production of matrix metalloproteinase-13 (MMP-13) *via* E-type prostaglandin (EP) receptor 1 (EP1) in human periodontal ligament cells [59]. It is considered that endogenous PGE_2_ may be involved in regulating the destruction of extracellular matrix components in periodontal lesions. However, exogenous PGE_2_ may act as an anti-inflammatory effect *via* the inhibitory prostanoid receptor(s) EP receptor [60]. Therefore, melatonin may prevent various oral diseases including periodontitis, even neoplastic diseases such as precancerous leukoplakia, lichen planus, and oral cancer [56].

**Figure 2:**
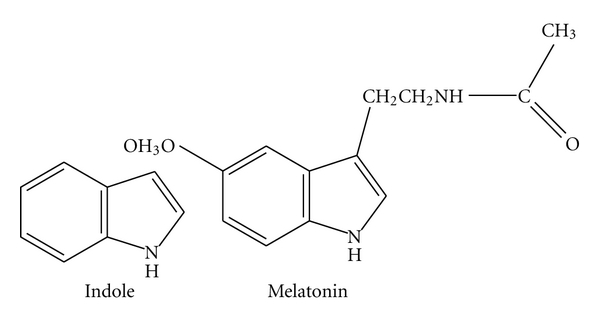
The chemical structures of melatonin and indole (Copyright *In Vivo*, Athens, Greece).

Therefore, to investigate whether COX-2 expression and nuclear factor kappa B (NF-*κ*B) activation in RAW264.7 cells stimulated by Pg fimbriae can be suppressed by melatonin, it is useful to consider the contribution of melatonin to not only the prevention of oral diseases, but also the regeneration of alveolar bone through stimulation of type I collagen fiber production and modulation of osteoblastic and osteoclastic activity mediated by cellular proteins. Fimbria-dependent activation of cell adhesion molecule expression in Pg-infected endothelial cells may play a role in the pathogenesis of systemic inflammatory diseases such as atherosclerosis, as well as periodontal diseases associated with this microorganism [61]. Thus, chronic infection induced by Pg that is linked to the initiation of periodontitis and atherosclerosis may be prevented by melatonin. Here, we present the results of our recent experimental study of COX-2 expression and NF-*κ*B activation in RAW264.7 cells stimulated by Pg fimbriae in the absence or presence of melatonin and indole [22], the latter being used as a negative control. The structures of melatonin and indole are shown in Figure 2. Melatonin at noncytotoxic concentrations was shown to significantly inhibit fimbria-induced COX-2 expression in RAW264.7 cells at both the mRNA and protein levels using Northern and Western Blotting, respectively. In contrast, the inhibitory effect of indole was not complete within a concentration range of 10–100 *μ*M. Furthermore, Western Blotting showed that melatonin strongly inhibited the production of COX-2, whereas indole at 100 *μ*M had only a weak inhibitory effect. These findings suggested that in RAW264.7 cells, melatonin exerted a markedly stronger inhibitory effect on COX-2 expression than indole [22].

**Figure 3:**
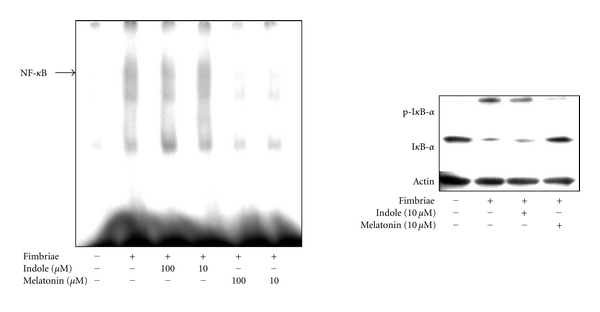
Inhibition of fimbria-stimulated NF-*κ*B activation by melatonin and indole in RAW264.7 cells. Left hand panel: inhibitory effects of melatonin and indole on fimbria-stimulated NF-*κ*B binding. The cells were pretreated for 30 min with or without the indicated doses of melatonin or indole and then incubated with or without fimbriae at 4 *μ*g/mL for 1 h. A gel mobility shift assay was performed with nuclear proteins and ^32^P-labeled oligonucleotide containing the NF-*κ*B consensus sequence. Three independent experiments were performed, and similar results were obtained. Right hand panel: inhibitory effects of melatonin and indole on phosphorylation-dependent proteolysis of fimbria-stimulated I*κ*B-*α*. The cells were pretreated for 30 min with or without melatonin or indole at 10 *μ*M and then incubated with or without fimbriae at 4 *μ*g/mL. Equal amounts of cell lysates were analyzed by Western Blotting after SDS-PAGE with phosphospecific anti-I*κ*B-*α* antibody, anti-I*κ*B-*α* antibody, or anti-*β*-actin antibody. Three independent experiments were performed, and similar results were obtained (Copyright *In Vivo*, Athens, Greece).

To clarify whether melatonin and indole are inhibitors of fimbria-stimulated NF-*κ*B, their inhibitory effects on the binding of NF-*κ*B to its consensus sequence in fimbria-stimulated RAW264.7 cells were investigated using EMSA. The fimbria-stimulated binding of NF-*κ*B was markedly inhibited by melatonin, whereas indole inhibited the binding only weakly (Figure 3). In addition, melatonin clearly inhibited both the phosphorylation and degradation of I*κ*B-*α* stimulated by the fimbriae. These findings suggest that melatonin is a potent inhibitor of fimbria-triggered cellular signaling in RAW264.7 cells.

Deng et al. previously reported that melatonin, but not tryptophan or serotonin, inhibited LPS or LPS plus IFN*γ*-induced COX-2 and iNOS transcriptional activation in RAW264.7 cells by suppressing p52 acetylation [58]. The most well-known derivative of indole is the amino acid tryptophan, the precursor of the neurotransmitter serotonin. Melatonin, serotonin, and tryptophan share a common tryptophan backbone. Among them, however, melatonin alone exhibited anti-inflammatory activity, suggesting the requirement of a melatonin side chain for inhibition of COX-2 and iNOS expression at both the protein and gene levels. The basic structure of melatonin comprises an indole ring with a methoxy group at position 5 (5-methoxy group) and an acylaminoethyl side*-*chain at position 3, whereas indole has no side chains (Figure 2). The two side chains on the ring may be important for inhibiting COX-2 and iNOS expression at the protein and gene levels.

Our findings suggest that melatonin significantly inhibits Pg fimbria-induced expression of the COX-2 gene through suppression of NF-*κ*B activation in RAW264.7 cells and thus may help prevent Pg-induced oral diseases and chronic infections in the body.

Korkmaz et al. have shown that melatonin efficiently protects biomolecules such as lipids, proteins, and nucleic acid from nitrooxidative stress [62], and García et al. have reported that melatonin prevents changes in microsomal membrane fluidity during lipid peroxidation, suggesting a cytoprotective effect [63]. We investigated the cytotoxicity of melatonin towards RAW264.7 cells and found that it was much lower than that of indole, possibly due to the lower hydrophobicity of the former [22]. Tan et al. have reported that the protective effect of melatonin on biomolecules may be related to its ability to scavenge reactive oxygen species (ROS) and reactive nitrogen species (RNS) [64]. Also, Antolín et al. have shown that melatonin exerts a cytoprotective effect by inhibiting aminolevulinate synthase (and thus porphyrin synthesis) and some prooxidant enzymes [65]. Furthermore, the radical-scavenging activity of melatonin towards alkyl and peroxy radicals is reportedly greater than that of indole [66]. Melatonin scavenges a variety of ROS and RNS, including the hydroxyl radical, hydrogen peroxide, singlet oxygen, nitric oxide, and the peroxynitrite anion [48]. The cytoprotective activity of melatonin may be derived from its high endogenous radical-scavenging activity, which in turn could explain its anti-inflammatory activity.

Recently, NSAID-like compounds have become a focus of attention with regard to the prevention of various chronic diseases, including oral diseases such as periodontitis and precancerous diseases such as lichen planus [19, 20, 67]. We previously investigated the antioxidative and anti-inflammatory activity of some natural and synthetic phenols, which are NSAID-like compounds possessing high antioxidative activity. We found that, in particular, *ortho*-dimer phenols, *bis*-BHA (3,3′-di-t-butyl-5,5′-dimethoxy-1,1′-biphenyl-2,2′diol), showed good radical-scavenging activity and also inhibited Pg fimbria-stimulated inflammatory cytokine expression and NF-*κ*B activation in RAW264.7 cells [68]. In addition, we have confirmed that magnolol and/or honokiol, which are hydroxylated biphenyl compounds isolated from *Magnolia officinalis*, inhibit the fimbria-stimulated COX-2 expression resulting from NF-*κ*B activation in the same cells (data not shown). These findings suggest that the anti-inflammatory activity of melatonin may be related to its potent radical-scavenging activity.

## 4. Conclusions

The inhibitory effect of FN on Pg fimbria-induced proinflammatory cytokines and that of melatonin on Pg fimbria-induced COX-2 and NF-*κ*B expression indicate that these bioactive compounds possess anti-inflammatory activity and may be applicable clinically for the prevention of oral diseases and chronic infections in the body induced by periodontopathic bacteria.
